# Construction of a diagnostic model and identification of effect genes for diabetic kidney disease with concurrent vascular calcification based on bioinformatics and multiple machine learning approaches

**DOI:** 10.3389/fmolb.2025.1609307

**Published:** 2025-10-14

**Authors:** Lili Huang, Wenjing Wu, Shenhui Lv, Danfang Deng, Xiaoqin Wang

**Affiliations:** ^1^ Clinical College of Chinese Medicine, Hubei University of Chinese Medicine, Wuhan, China; ^2^ Department of Nephrology, Affiliated Hospital of Hubei University of Chinese Medicine, Hubei Provincial Hospital of Traditional Chinese Medicine, Wuhan, China; ^3^ The First Clinical Medical School, Hubei University of Chinese Medicine, Wuhan, China; ^4^ Hubei Key Laboratory of Theory and Application Research of Liver and Kidney in Traditional Chinese Medicine, Hubei Provincial Hospital of Traditional Chinese Medicine, Wuhan, China

**Keywords:** diabetic kidney disease, vascular calcification, bioinformatics, multiple machine learning, diagnostic model

## Abstract

**Objective:**

This study aims to construct a diagnostic model for diabetic kidney disease (DKD) with concurrent vascular calcification (VC) using bioinformatics combined with machine learning approaches and to explore the potential underlying mechanisms.

**Methods:**

RNA sequencing (Bulk-seq) data of DKD and VC from various species were obtained from the Gene Expression Omnibus (GEO) database, and relevant datasets were integrated. Differential analysis of the DKD and VC datasets was performed using the limma package and weighted gene co-expression network analysis (WGCNA) in *R* (Ver. 4.3.3). Common differentially expressed genes (DEGs) and module genes were identified. Multiple machine learning algorithms were applied to select the optimal diagnostic model and identify hub genes, including LASSO regression, Random Forest, Gaussian Mixture Model (GMM), and Support Vector Machine-Reference (SVM-REF). Diagnostic performance was evaluated using the receiver operating characteristic (ROC) and precision-recall (PR) curves. Gene ontology (GO), Kyoto Encyclopedia of Genes and Genomes (KEGG), gene set enrichment analysis (GSEA), and Cibersort immune infiltration analysis were conducted to explore the potential shared pathological mechanisms between DKD and VC.

**Results:**

A total of five coDEGs (JUN, KCND3, HIP1, PTGDS, SLC22A17) were identified in our study. Among these three genes, JUN, PTGDS, and SLC22A17 demonstrated the best performance (validation group AUC: 1, test group AUC: 0.897) in the diagnostic model constructed by the SVM-REF machine learning method. Functional enrichment analysis of hub genes mainly involved biological processes such as inflammation, osteoblastic differentiation, apoptosis, and ferroptosis. Immune infiltration analysis revealed that in DKD patients, the expression levels of Memory B Cells, CD8^+^ T cells, M1 macrophages, M2 macrophages, resting dendritic cells, and resting mast cells were increased. In contrast, the expression of follicular helper T cells, activated mast cells, and neutrophils decreased relatively.

**Conclusion:**

This study suggests that JUN, PTGDS, and SLC22A17 may be potential biomarkers for DKD with VC, involving immune, metabolic, and inflammatory processes. These findings provide new targets for early diagnosis of DKD with VC and offer a novel perspective for applying bioinformatics combined with machine learning in discovering diagnostic biomarkers for diseases.

## 1 Introduction

Diabetic Kidney Disease (DKD) is a common microvascular complication of diabetes in clinical practice. According to the 2019 Annual Data Report on Kidney Diseases in China, DKD has become the leading cause of hospitalization among patients with Chronic Kidney Disease (CKD) in China, accounting for 27%. A survey by the Global Burden of Disease Collaborative Organization for Chronic Kidney Disease showed that as of 2017, organ failure caused by CKD directly led to 1.2 million deaths, and the number of deaths from cardiovascular diseases caused by CKD reached 1.36 million (Ding et al., 2023). Vascular calcification (VC), an independent predictor of cardiovascular diseases, has been proven to be an independent risk factor related to the (all-cause) mortality rate of CKD and ESRD patients (Jung and Yoo, 2022; Amaya-Garrido et al., 2023). Clinical studies have shown that about 50%–90% of CKD patients in 3 - 5D stages have symptoms of VC (Martin et al., 2024; Buckley et al., 2023). Therefore, the keys to the prevention and treatment of DKD have become improving the diagnostic efficiency of DKD, controlling risk factors in the early stage of the disease, intervening in the process of VC, and preventing and treating cardiovascular complications.

VC in DKD is a complex pathological process. Metabolic abnormality of DKD patients is one of the key factors promoting VC (Lanzer et al., 2014). Persistent high blood glucose levels can damage the function of vascular endothelial cells, promote oxidative stress reactions and the release of inflammatory mediators (Nieves-Cintrón et al., 2021; Zhao et al., 2021). At the same time, as the renal function of DKD patients declines, the blood phosphorus level rises, and the deposition of phosphates and calcium salts in the blood vessel wall leads to vascular stiffness and elasticity reduction, all of which are key factors promoting VC (Salam et al., 2021; Qin et al., 2025). As DKD progresses to end-stage renal disease, patients who need dialysis treatment are more likely to develop severe VC problems (Thakker et al., 2025). This is not only because the metabolic abnormality mentioned above is more severe but also related to the dialysate containing calcium used during dialysis, which may directly promote the accumulation of calcium in the body. In summary, DKD promotes the occurrence and development of VC through multiple mechanisms, which in turn increases the burden on the cardiovascular system, forming a vicious cycle.

In recent years, bioinformatics and machine learning technologies have shown great potential in medical research, especially in identifying disease diagnostic markers (Leiherer, 2024). For DKD and VC, two complex and interrelated pathological states, bioinformatics and machine learning technologies can efficiently identify valuable biomarkers from a large amount of biological data. Although numerous studies have shown that the occurrence and development of VC are closely related to DKD, the common potential biomarkers of these two diseases remains elusive. In this study, bioinformatics methods were used to integrate the data of Bulk-seq of whole samples from different species. On this basis, machine learning was used to screen out the key biomarkers for diagnosing DKD with VC and to explore the internal relationship and possible biological mechanisms between them. This might provide a basis for early disease diagnosis, risk assessment, and treatment strategies.

## 2 Materials and methods

**FIGURE 1:**
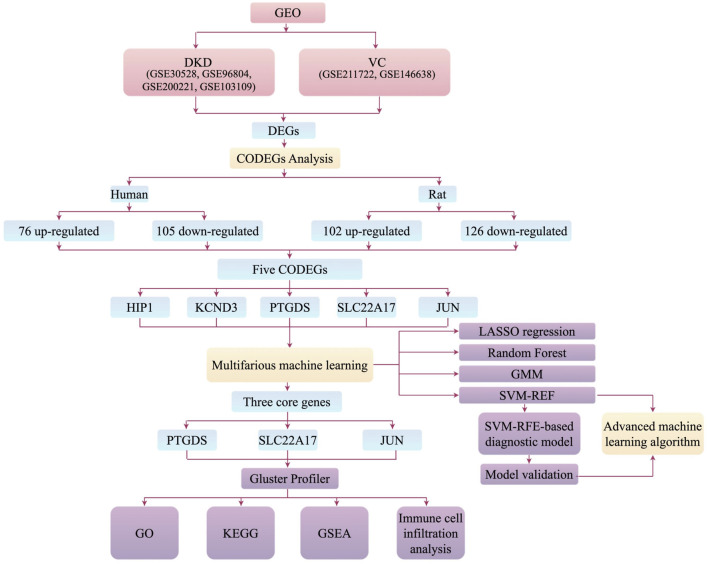
Flow chart of research design.

The overall design of the study is illustrated in Figure 1.

### 2.1 Data sources and processing

The Bulk-seq data related to DKD and VC were sourced from the GEO database (https://www.ncbi.nlm.nih.gov/geo/), which has already undergone standardization. Nine DKD samples and thirteen control samples were obtained from GSE30528, and forty-one DKD samples and twenty control samples were obtained from GSE96804, all from human glomerular tissues. Three human VC samples and three control samples were retrieved from GSE211722. Additionally, four DKD and four control samples were sourced from GSE200221, and two DKD samples and three control samples were retrieved from GSE103109, all derived from rats. Furthermore, nine rat VC samples and five control samples were collected from GSE146638.

We applied the ComBat algorithm and used the sva package in the R language to perform batch effect correction on GSE96804 and GSE30528. This method adjusts the systematic differences between different batches through a Bayesian framework, while maximizing the retention of the biological variation signals in the data. The core principle is to perform robust correction of the expression levels of each gene in different batches while controlling the batch effect. While batch effect correction and merging were applied to GSE200221 and GSE103109 (Supplementary Figure S1), the final datasets were as follows: for human Bulk-seq: DKD samples (n = 50), DKD control samples (n = 33), VC samples (n = 3), and VC control samples (n = 3). For rat Bulk-seq: DKD samples (n = 6), DKD control samples (n = 7), VC samples (n = 9), and VC control samples (n = 5).

### 2.2 Identification of differentially expressed genes (DEGs) and overlapping genes

The NCBI HomoloGene database was used to uniformly convert rat genes into human homologous gene symbols to ensure comparability of cross-species genes. Differential expression analysis of the DKD and VC datasets was sequenced using the limma R package in (Ver. 4.3.3), with a significance threshold of *p* < 0.05 and log2 (fold change) > 0. The DEGs were then visualized using the Enhanced Volcano package in *R* software, and those that were consistently upregulated or downregulated across human, rat, DKD, and VC datasets were considered common genes. The overlapping DEGs across the four groups were visualized using Venn diagrams and displayed through STRING (https://cn.string-db.org/) for further visualization.

### 2.3 Machine learning

A stratified sampling strategy was employed, using the sample function in R to randomly select 70% of the samples from the GSE96804 dataset as the training group for machine learning model development; the remaining 30% were retained as the validation group. Finally, the GSE30528 dataset was used as an external testing group to further evaluate the model’s performance and diagnostic ability on an independent dataset. Subsequently, several machine learning algorithms, including LASSO regression, Random Forest, Gaussian Mixture Model (GMM), and Support Vector Machine with reference (SVM-REF), were utilized to identify hub genes. The Receiver Operating Characteristic (ROC) curves of these genes were plotted.

This study ensures the robustness of the model through structured parameter configuration and multi-level validation strategies. LASSO regression employs 10-fold cross-validation (nfolds = 10) to automatically determine the optimal regularization strength λ (lambda.min), thereby achieving Feature compression and overfitting suppression. The random forest algorithm fixes the number of decision trees (ntree = 100) and recursively evaluates the performance of feature subsets via 10-fold stratified cross-validation (method = “LGOCV,” number = 10), terminating when the highest accuracy on the validation set is achieved. SVM-RFE iteratively eliminates the 10% of features with the lowest weights in each round (halve.above = 100) and dynamically screens for optimal feature combinations through 5-fold cross-validation (k = 5) until the combination with the lowest error rate is identified. The Gaussian mixture model performs logistic regression on all possible gene combinations and selects the group with the highest AUC value based on clustering analysis.

The best-performing diagnostic model was selected as the hub genes set. Using Python software, the Cat Boost advanced machine learning algorithm was applied to calculate the Area Under the Curve (AUC) values of these hub genes, and the corresponding ROC curve was plotted. The importance of these hub genes was then visualized. Linear Discriminant Analysis (LDA) and Quadratic Discriminant Analysis (QDA) algorithms from the MASS package in R were employed to construct models with the hub genes and assess their diagnostic performance, generating corresponding heatmaps. Additionally, an artificial neural network was built for the testing group using the neural net package in R, with a default of 5 hidden layer neurons and an output layer for DKD and control groups. Deep learning methods were applied to further validate the importance of the hub genes.

### 2.4 Functional enrichment analysis

Based on the hub genes, Gene Ontology (GO) functional enrichment analysis and Kyoto Encyclopedia of Genes and Genomes (KEGG) pathway enrichment analysis were performed using the ClusterProfiler package, with a significance threshold of *p* ≤ 0.05. The results were visualized through bar plots. The most significant genes among the hub genes were selected for single-gene Gene Set Enrichment Analysis (GSEA), and the corresponding enrichment score plot was generated.

### 2.5 Immune cell infiltration analysis

The cybersport algorithm from the IOBR package in R was utilized to assess the types and quantities of immune cells in the GSE96804 dataset. The proportions of immune cells in each sample were visualized using the ggplot2 package. Additionally, boxplots of immune cell distributions across multiple groups were generated using the boxplot function. At the same time, either the T-test or the Wilcoxon rank-sum test was employed to compare inter-group differences. A statistically significant difference was indicated when *p* < 0.05.

## 3 Results

### 3.1 DEGs analysis and identification of common genes across species

#### 3.1.1 DEGs and visualization for DKD and VC data sets of different species

**FIGURE 2:**
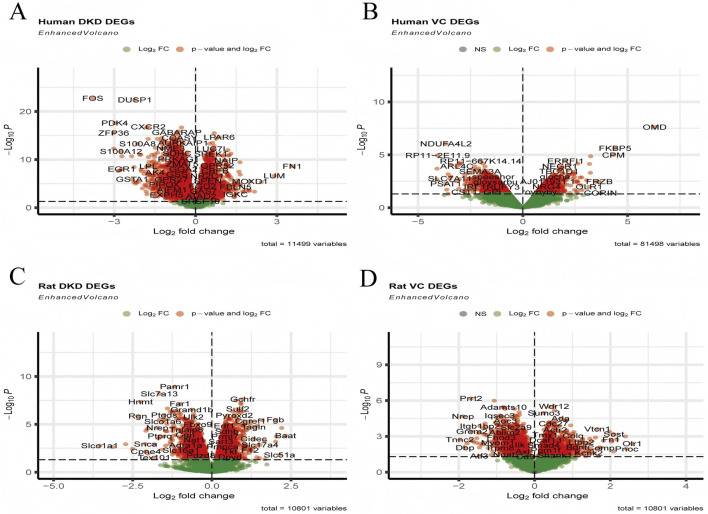
Visualization of DEGs. **(A)** Volcano plot of human DKD, **(B)** Volcano plot of human VC, **(C)** Volcano plot of rat DKD, and **(D)** Volcano plot of rat VC.

In human DKD, a total of 2,541 upregulated and 3,684 downregulated DEGs were identified. In comparison, 1,515 upregulated and 2,043 downregulated DEGs were enriched in human VC. There were 1,590 upregulated and 1,555 downregulated DEGs in rat DKD, and 1,060 upregulated and 1,094 downregulated DEGs were observed in rat VC (Figures 2A–D).

#### 3.1.2 Identification of cross-species overlapping DEGs between DKD and VC and analysis of gene interaction networks

**FIGURE 3:**
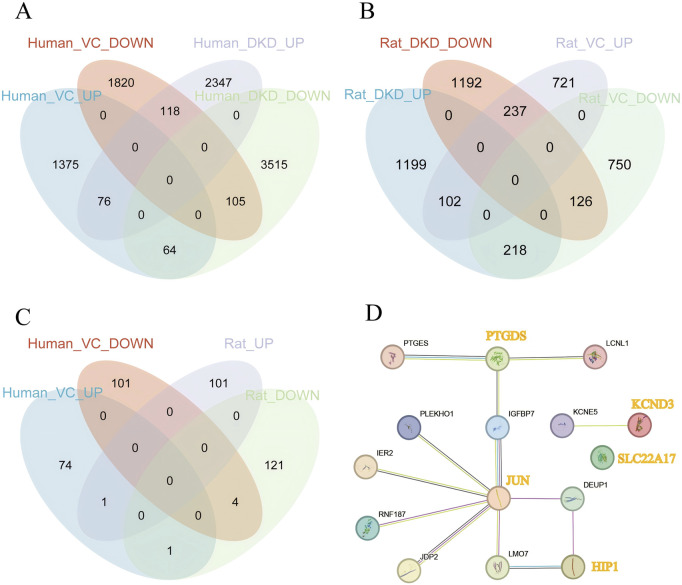
Identification of Overlapping Genes. **(A)** Identification of overlapping genes in human DKD and VC, **(B)** Identification of overlapping genes in rat DKD and VC, **(C)** Identification of overlapping genes between human and rat DKD and VC, **(D)** STRING interaction network of overlapping genes and their associated genes.

As shown in Figures 3A–D, in human DKD-VC, a total of 76 upregulated DEGs and 105 downregulated DEGs were obtained (Figure 3A); in the rat DKD-VC model, 102 up-regulated DEGs and 126 down-regulated DEGs were identified. Finally, five overlapping DEGs were obtained by taking the intersection of the two sets: HIP1, KCND3, JUN, PTGDS, and SLC22A17. The STRING relationship diagram shows the connections between these or their associated genes.

### 3.2 Hub genes screening and diagnostic model construction based on machine learning

#### 3.2.1 Screening of hub genes using multiple machine learning algorithms

**FIGURE 4:**
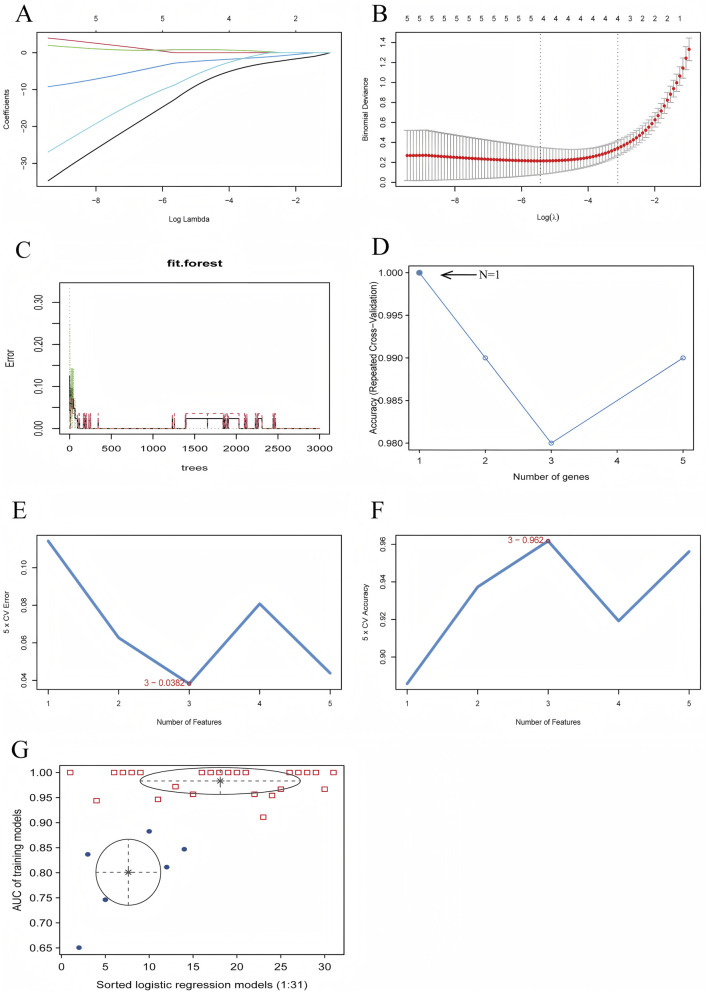
Diagnostic Model Construction Using Machine Learning Algorithms. **(A)** LASSO regression coefficient plot, **(B)** LASSO regression cross-validation plot, **(C)** Random forest prediction accuracy plot, **(D)** Random forest accuracy plot, **(E)** SVM-RFE error rate plot, **(F)** SVM-RFE accuracy plot, **(G)** GMM regression model evaluation plot.

Using LASSO regression analysis, we have identified four key genes—JUN, KCND3, PTGDS, and SLC22A17—when the lambda value reached its minimum. Figures 4A,B present the regression coefficients and the outcomes of regression cross-validation, respectively, which provide the performance and reliability of the model. Further, the random forest algorithm found that when the hub genes was identified as JUN, the accuracy rate was the highest, and the model’s prediction accuracy was visualized (Figures 4C,D). Subsequently, an SVM-RFE algorithm was used to construct a predictive model, achieving the lowest error rate of 0.038 and the highest accuracy of 0.962 when the model included three key genes: JUN, PTGDS, and SLC22A17 (Figures 4E,F). Moreover, through Gaussian Mixture Model (GMM) analysis, we identified four hub genes—JUN, HIP1, PTGDS, and SLC22A17—when the ROC curve AUC value reached its highest point of 1.00, and the AUC values for each regression model were visualized in Figure 4G.

#### 3.2.2 Verification of hub genes and performance evaluation of the optimal diagnostic model

**FIGURE 5:**
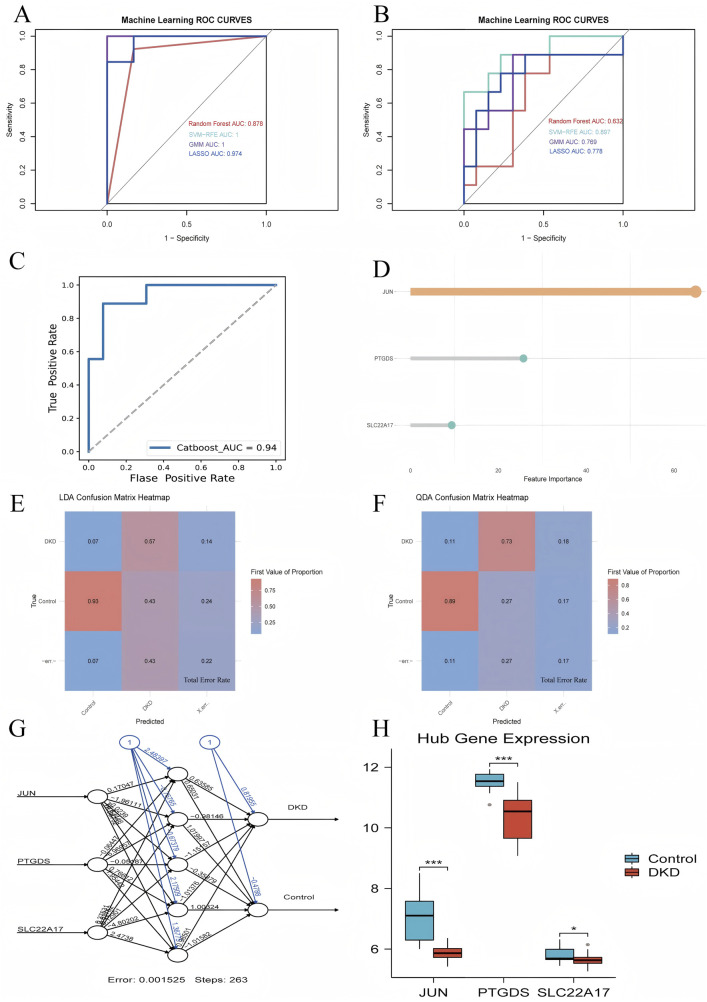
Validation of hub genes. **(A)** ROC curve for the validation set using four machine learning methods, **(B)** ROC curve for the test set using four machine learning methods, **(C)** ROC curve for the CatBoost model, **(D)** Gene weight plot for the CatBoost model, **(E)** LDA confusion matrix heatmap, **(F)** QDA confusion matrix heatmap, **(G)** Artificial neural network model plot (numbers on the lines represent the predicted weights), **(H)** Group comparison plot of hub genes expression levels.

We constructed diagnostic models using machine learning methods, which were validated with validation and test sets, with the corresponding ROC curves drawn and AUC values calculated. The AUC values of the RF, SVM-RFE, LASSO, and GMM validation groups were 0.878, 1, 1, and 0.974, respectively, while the AUC values of the test group were 0.632, 0.897, 0.769, and 0.778, respectively (Figures 5A,B). Based on these results, we selected the diagnostic model (JUN, PTGDS, SLC22A17) constructed by the SVM-RFE, which exhibited the best prediction effect for the Catboost advanced machine learning algorithm. The ROC curve revealed that its AUC value was 0.94, demonstrating its diagnostic accuracy and reliability (Figures 5C,D). Subsequently, the LDA and QDA algorithms were used to further predict this model’s error rate (Figures 5E,H). Among them, the LDA algorithm displayed that the error rates predicted by this model for the control and DKD groups were 0.07 and 0.43, respectively, with an overall error rate of 0.22. Using the QDA algorithm, it has been found that the error rates for the control and DKD groups were 0.11 and 0.27, respectively, resulting in an overall error rate of 0.17. Indicates a reasonably good model performance in distinguishing between the two groups. Surprisingly, the artificial neural network graph showed that the model ran for 263 steps, and the error rate was only 0.001525. Additionally, in the training group, a comparison chart of the expression levels of hub genes by group was displayed, showing that the hub genes were expressed at a lower level in DKD patients compared to the control group.

### 3.3 Functional enrichment analysis

**FIGURE 6:**
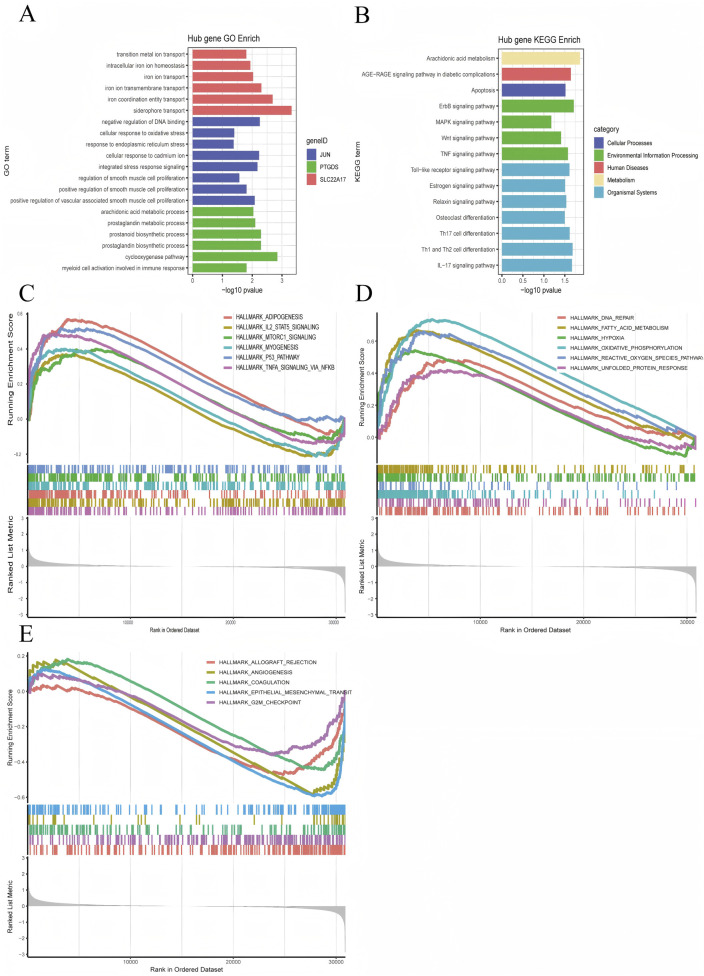
Visualization of Enrichment Analysis Results. **(A)** GO enrichment analysis results for the hub genes, **(B)** KEGG enrichment analysis results for the hub genes, **(C–E)** JUN GSEA Hallmark enrichment score plots.

In order to better explain the biological functions of the common genes and the biological functions of the shared genes, GO and KEGG pathway annotations were used to describe and analyze the coDEGs screened in the above steps. GO enrichment analysis revealed that the hub genes were significantly enriched in processes such as promoting or enhancing vascular smooth muscle cell proliferation, integrated stress response, cellular response to cadmiumions, arachidonic acid metabolism, prostaglandin metabolism, activation of myeloid cells, and ironion transport (Figure 6A). The results of the KEGG enrichment analysis showed that the hub genes were significantly enriched in pathways including the TNF signaling pathway, Wnt signaling pathway, MAPK signaling pathway, Toll-like receptor signaling pathway, IL-17 signaling pathway, apoptosis, AGE-RAGE signaling pathway, and arachidonic acid metabolism (Figure 6B).

Furthermore, we conducted a GSEA analysis on the hub gene JUN, which performed the best. According to the JUN expression level (whether it is higher than the median), we divided the DKD patients into the JUN high-expression group and the low-expression group. We then performed GSEA enrichment analysis using the hallmark gene sets, after plotting the GSEA enrichment analysis score graphs for the top 12 positive values and the five negative values according to the absolute value of NES, as displayed in Figures 6C–E. The JUN high-expression group was mainly enriched in adipogenesis, IL-2/STAT5 signaling pathway, mTORC1 signaling pathway, myogenesis, p53 signaling pathway, TNF-α via NF-κB signaling pathway, DNA repair, fatty acid metabolism, hypoxia, oxidative phosphorylation, ROS pathway, and unfolded protein response, etc.; the JUN low-expression group was mainly enriched in allograft rejection, angiogenesis, coagulation, epithelial-mesenchymal transition.

### 3.4 Assessment and visual analysis of the immune infiltration

**FIGURE 7:**
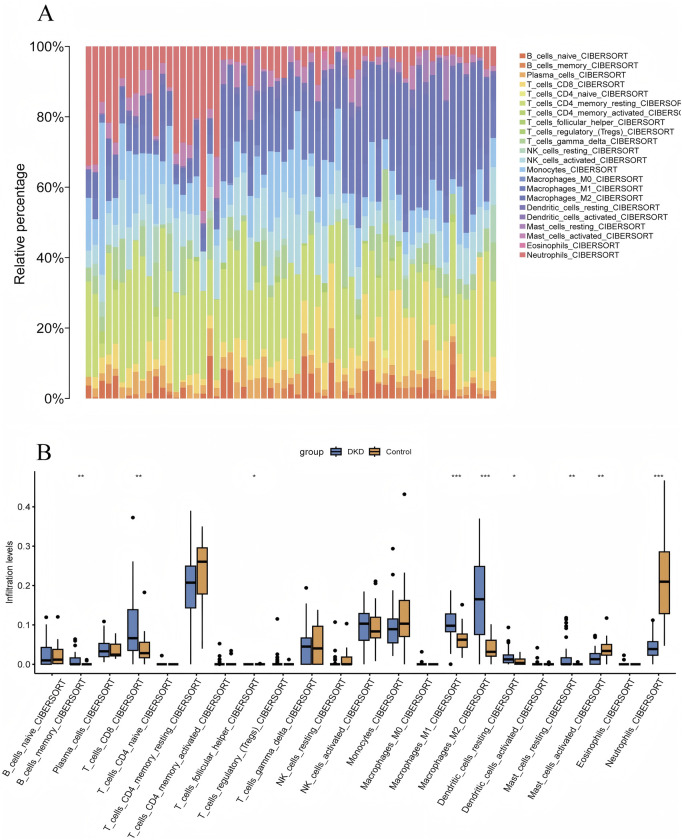
Visualization of Immune Infiltration Analysis Results. **(A)** CIBERSORT immune infiltration rainbow proportion plot, **(B)** CIBERSORT multi-group boxplot.

The algorithm of ssGSEA was used to quantify the distribution and relative proportions of the relative infiltration levels of 22 immune cells from the GSE96804 datasets. As seen in Figures 7A,B, a significant difference in the distribution and proportion of major immune cell types in DKD and VC patients and healthy controls. Including memory B cells, CD8^+^ T, activated NK cells, M1 macrophage cells, M2 macrophage cells, resting dendritic cells, as well as resting mast cells were significantly upregulated in DKD patients. In contrast, the activated mast and neutrophil cells were significantly downregulated in DKD patients. There was no significant difference between naive B cells, Plasma cells, activated CD4 T cells, helper follicular T cells, tregs, regulatory T cells, M0 macrophage cells, and activated dendritic cells. The results could help us better evaluate the connection between the immune pathways for the DKD-VC between diseases and healthy controls.

## 4 Discussion

Integrating bioinformatics with machine learning algorithms is becoming increasingly common in exploring new genes, potential diagnostic and prognostic biomarkers, and possible therapeutic targets using big data (Li et al., 2022; Shi et al., 2025). Our study systematically analyzed the interactions among immune, metabolic, and inflammatory processes, identifying actionable biomarkers and molecular pathways. These findings can provide valuable insights into the comorbidity of DKD-VC.

Growing evidence has suggested that DKD and VC have multiple interactions regarding mineral metabolism disorders (Winiarska et al., 2021), immune response (Zhang et al., 2025) and inflammation (Fatima et al., 2024), oxidative stress (Qin et al., 2025), and cell phenotypic transformation (Lanzer et al., 2025). It is mainly because both conditions are accompanied by similar risk factors such as hypertension and dyslipidemia (Kadowaki et al., 2022). Among them, a persistent hyperglycemia state was analogous to considered the starting point for vascular lesions, and chronic low-grade inflammatory response and the decline in renal function caused by DKD are accelerators (Bavanandan et al., 2025). Hyperglycemia can damage the tiny blood vessels in the kidneys, accompanied by a decrease in glomerular filtration rate, as the kidneys are the core organ for regulating the balance of calcium, phosphorus, vitamin D, and parathyroid hormone (PTH) (Lee et al., 2020). Once the renal function is impaired, a series of mineral and bone metabolism abnormalities will occur, resulting in the’ active deposition of hydroxyapatite crystals in the middle layer of the vascular wall, ultimately forming VC (Huang et al., 2024; Liu et al., 2021).

Through multiple machine learning approaches and model validation, this study finally identified three biomarkers with the highest accuracy and sensitivity: JUN, PTGDS, and SLC22A17. Unlike others, as a core component of the activator protein-1 (AP-1) transcription factor family, JUN can directly bind to the promoter regions of numerous osteogenic-related genes such as BMPs, BMP-2, and OPN, or interact with the key master regulatory factor RUNX2 (Chen et al., 2018), jointly activating the complete osteogenic gene expression program, thereby promoting vascular calcification (Tang et al., 2021; Lian et al., 2025). At the same time, after J-Jun is activated, it may also upregulate the expression of specific mineral regulatory proteins (such as the matrix calcification inhibitor), further exacerbating VC (Guo et al., 2025; Zhou et al., 2021). In our study, it is noteworthy that JUN serves as a hub genes, and based on the Catboost algorithm, it is shown that JUN has the highest importance compared to other hub genes. We have reason to speculate that JUN’s abnormal expression or activation may be regarded as a potential driving factor for VC in patients with DKD.

PTGDS, a glutathione-independent prostaglandin synthase, exhibits dual roles as a neuroregulatory mediator and inflammatory modulator (Martín-Vázquez et al., 2023). Transcriptomic profiling reveals PTGDS-associated differential genes are enriched in arachidonic acid metabolism, driving inflammatory mediator production through prostaglandin-dependent cascades (Wang et al., 2021). Clinical studies have demonstrated that SGLT2 inhibitors also downregulate PTGDS-containing protein complexes (among 19 targets) and mitigate TGF-β-induced epithelial-mesenchymal transition in diabetic nephropathy (Ahluwalia et al., 2023). It is the predominant expression in adipose tissue and is associated with cardiovascular endpoints such as coronary atherosclerosis and heart failure (Yang H. H. et al., 2023). As a result, PTGDS is also suggested as a metabolic-inflammatory nexus during DKD-VC progression (Tang et al., 2021).

The role of SLC22A17 in DKD combined with VC may be related to its functions in iron homeostasis and apoptosis regulation (Khanal et al., 2025; Li et al., 2024). The transporter protein encoded by SLC22A17 participates in the regulation of iron ions within cells (Jaberi et al., 2021), and an iron homeostasis disorder can exacerbate oxidative stress and trigger chronic inflammation (Galy et al., 2024), which further disrupts iron metabolism and leads to abnormal SLC22A17 function, increasing the generation of ROS, which is a causal factor of damaging vascular endothelial cells and VSMCs(Khanal et al., 2025). However, the complex mechanism of vascular calcification involves the synergistic action of multiple genes, pathways, and targets. These genes do not work independently but form a complex immune-inflammation-cell-vascular interaction network, thereby triggering the onset and progression of calcification.

Via GO and KEGG enrichment analysis of coDEGs from various species, it was discovered that in the DK-VC, signaling pathways, such as TNF, Wnt, MAPK, and Toll-like receptor (Wu et al., 2025), were upregulated, from which the inflammatory cascade emerges as a central pathogenic mechanism. As outlined earlier, high glucose and chronic inflammatory states would activate inflammatory signaling pathways such as Toll-like receptors and TNF (Rostoff et al., 2024), leading to the accumulation of ROS and intensified oxidative stress (Luna-Marco et al., 2024), causing the transformation of VSMCs from a contractile type to an osteogenic type. In addition, the JUN and MAPK pathways regulate the expression of pro-inflammatory genes and apoptosis-related proteins, amplifying the apoptotic signal, thereby further triggering the integrity of vascular wall cells (Liu et al., 2023; He et al., 2021). Apoptotic VSMCs create a nucleation microenvironment for hydroxyapatite crystal deposition through membrane vesicle release and phosphatidylserine exposure, mechanistically contributing to the initiation phase of vascular calcification. The Wnt signaling pathway is abnormally activated in the osteoblast-like calcification process, promoting the mineralization of VSMCs (Moncla et al., 2023). Overall, inflammation, apoptosis, and osteogenic transdifferentiation are engaged in DKD combined with VC, leading to vascular wall calcification.

In this investigation, CIBERSORT analysis revealed that, M1, M2 macrophages, resting dendritic cells, and resting mast cells were significantly highly expressed in DKD patients. Notably, macrophages have been proven to directly participate in regulating the osteogenic transdifferentiation of VSMCs (Bai et al., 2025). Macrophages can alter their polarity, phenotype, or release functional substances such as tumor necrosis factor-α (TNF-α) as an adaptive response mechanism when exposed to changes in the tissue microenvironment (Park et al., 2025; Baba et al., 2024). What is more, they can also upregulate the expression of osteogenic-related genes (such as Runx2, BMP2) (Dong et al., 2025), thereby promoting the osteogenic transformation of VSMC and inhibiting its contractile phenotype. While M2-type macrophages, despite having a role in repairing tissues, can also contribute to fibrosis in the case of long-term chronic inflammation, and may also promote the osteogenic transdifferentiation of vascular smooth muscle cells, causing calcium ions to deposit in the vascular wall (Chen et al., 2025). Besides, after releasing tryptase and chymase, mast and dendritic cells can degrade the extracellular matrix, changing the local microenvironment and providing a site for VSMC calcification (Yang Y. et al., 2023). The results of this study have provided strong evidence that, in the context of DKD disease, the infiltration and activation of specific immune cells (such as macrophages M1, M2, resting dendritic cells, and resting mast cells) may be highly influenced by inflammation and changes in the vascular microenvironment.

## 5 Conclusion

**FIGURE 8:**
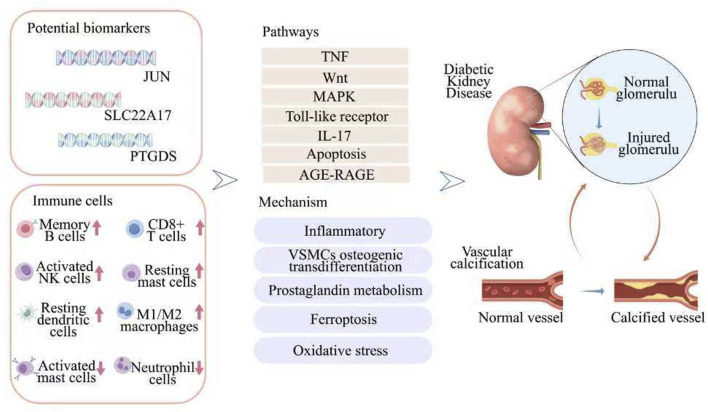
A flow diagram showing that multiple bioinformatics analysis identified potential biomarkers for predicting DKD-VC.

This work ultimately identified JUN, PTGDS, and SLC22A17 as potential diagnostic markers for DKD-VC. It also highlighted their interactions in various cellular processes such as inflammation, osteogenic differentiation, and immune processes (Figure 8). Moreover, it offers important insights into this condition’s clinical early diagnosis and intervention. In the future, after in-depth studies on the functions and mechanisms of these genes, combined with experimental verification, we expect to formulate precise treatment strategies to delay the progression of vascular calcification and improve the prognosis and quality of life of patients.

## Data Availability

The datasets presented in this study can be found in online repositories. The names of the repository/repositories and accession number(s) can be found below: https://www.ncbi.nlm.nih.gov/geo/, GSE30528, GSE96804, GSE211722.
