# Supplementary figures and images for: Construction of a diagnostic model and identification of effect genes for diabetic kidney disease with concurrent vascular calcification based on bioinformatics and multiple machine learning approaches
Source: Front Mol Biosci. 2025 Oct 14;12:1609307. doi: 10.3389/fmolb.2025.1609307 (PMC12558819; doi:10.3389/fmolb.2025.1609307)

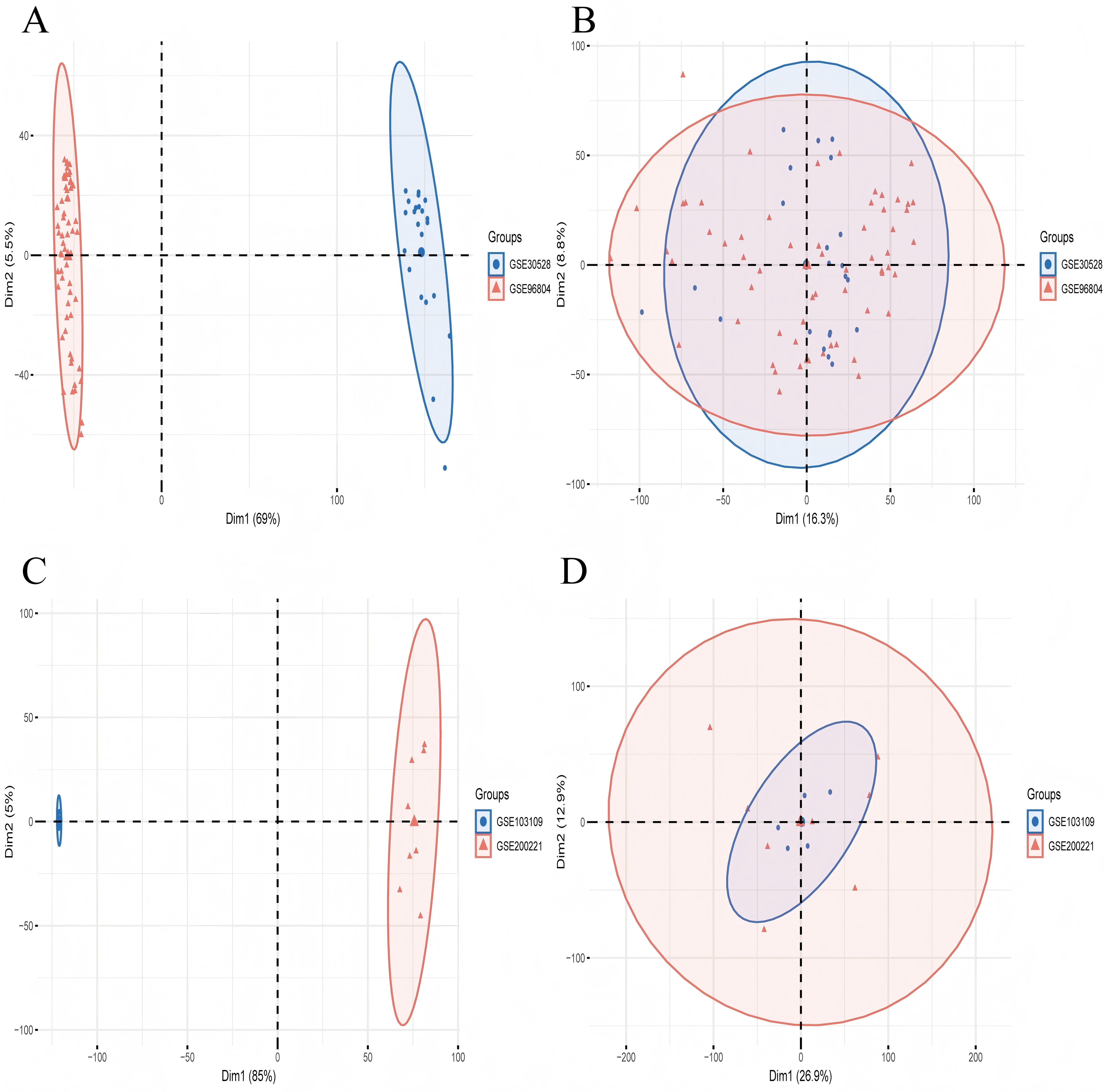

Supplement: Supplementary file 1 [file Image1.jpeg]
